# Histone acetylation and histone deacetylase activity of magnesium valproate in tumor and peripheral blood of patients with cervical cancer. A phase I study

**DOI:** 10.1186/1476-4598-4-22

**Published:** 2005-07-07

**Authors:** Alma Chavez-Blanco, Blanca Segura-Pacheco, Enrique Perez-Cardenas, Lucia Taja-Chayeb, Lucely Cetina, Myrna Candelaria, David Cantu, Aurora Gonzalez-Fierro, Patricia Garcia-Lopez, Pilar Zambrano, Carlos Perez-Plasencia, Gustavo Cabrera, Catalina Trejo-Becerril, Enrique Angeles, Alfonso Duenas-Gonzalez

**Affiliations:** 1 Unidad de Investigacion Biomedica en Cáncer, Instituto Nacional de Cancerología/Instituto de Investigaciones Biomédicas (INCan/IIB), Universidad Nacional Autonoma de Mexico (UNAM), Mexico City. Mexico; 2 Division of Clinical Research, Instituto Nacional de Cancerología (INCan), Mexico City, Mexico; 3 Laboratorio de Química Medicinal FES-Cuautitlán, UNAM, Mexico

## Abstract

**Background:**

The development of cancer has been associated with epigenetic alterations such as aberrant histone deacetylase (HDAC) activity. It was recently reported that valproic acid is an effective inhibitor of histone deacetylases and as such induces tumor cell differentiation, apoptosis, or growth arrest.

**Methods:**

Twelve newly diagnosed patients with cervical cancer were treated with magnesium valproate after a baseline tumor biopsy and blood sampling at the following dose levels (four patients each): 20 mg/kg; 30 mg/kg, or 40 mg/kg for 5 days via oral route. At day 6, tumor and blood sampling were repeated and the study protocol ended. Tumor acetylation of H3 and H4 histones and HDAC activity were evaluated by Western blot and colorimetric HDAC assay respectively. Blood levels of valproic acid were determined at day 6 once the steady-state was reached. Toxicity of treatment was evaluated at the end of study period.

**Results:**

All patients completed the study medication. Mean daily dose for all patients was 1,890 mg. Corresponding means for the doses 20-, 30-, and 40-mg/kg were 1245, 2000, and 2425 mg, respectively. Depressed level of consciousness grade 2 was registered in nine patients. Ten patients were evaluated for H3 and H4 acetylation and HDAC activity. After treatment, we observed hyperacetylation of H3 and H4 in the tumors of nine and seven patients, respectively, whereas six patients demonstrated hyperacetylation of both histones. Serum levels of valproic acid ranged from 73.6–170.49 μg/mL. Tumor deacetylase activity decreased in eight patients (80%), whereas two had either no change or a mild increase. There was a statistically significant difference between pre and post-treatment values of HDAC activity (mean, 0.36 vs. 0.21, two-tailed *t *test *p *< 0.0264). There was no correlation between H3 and H4 tumor hyperacetylation with serum levels of valproic acid.

**Conclusion:**

Magnesium valproate at a dose between 20 and 40 mg/kg inhibits deacetylase activity and hyperacetylates histones in tumor tissues.

## Background

The development of cancer has been associated with epigenetic alterations such as deregulation of DNA methylation and aberrant histone deacetylase (HDAC) activity [1]. These epigenetic phenomena co-participate in regulation of gene transcription: for instance, histone deacetylases which deacetylate histone core tails are recruited by DNA methyltransferases and methyl-binding proteins, which in turn lead to tighter chromatin packaging, reducing access of transcriptional factors to DNA [2,3].

HDACs are seen as a potential target for cancer treatment. HDAC inhibition has been reported to induce tumor cell differentiation, apoptosis, or growth arrest, depending on the experimental system [4-7]. Previous studies have shown that histone acetylation can increase the efficiency of several anticancer drugs targeting the DNA [8-11]; studies also shown that HDAC activity inhibitors enhance *in vitro *sensitivity of tumor cells to radiation [12-14]. However, a significant impediment in targeting HDAC has been lack of a clinically applicable HDAC inhibitor.

Valproic acid, an 8-carbon, branched-chained fatty acid, is a well-known and effective antiepileptic drug [15]. Its pharmacologic effects involve a variety of mechanisms including increased gamma-amino butyric acid (GABA)-ergic transmission, reduced release and/or effects of excitatory amino acids, blockade of voltage-gated sodium channels, and modulation of dopaminergic and serotoninergic transmission [16]. Because of its effectiveness, good tolerability, and oral bioavailability, valproic acid is widely used as a chronic anti-convulsant therapy.

It was recently reported that valproic acid is an effective inhibitor of histone deacetylases at concentrations well within the therapeutic range used for epilepsy. As such, valproic acid relieves repression of transcription factors that recruit histone deacetylases and activates transcription from diverse promoters. Valproic acid causes hyperacetylation of the N-terminal tails of histones H3 and H4 *in vitro *and *in vivo*. It inhibits HDAC activity, most probably by binding to the catalytic center and thereby blocking substrate access [17,18].

In contrast to other HDAC inhibitors, valproic acid has a good tolerability and safety profile as demonstrated by 35 years of use as a chronic therapy for epileptic disorders. It has a serum half-life of 9–18 h and is administered orally [15,16]; however, the dose needed for observing its HDAC inhibitory activity in tumors of cancer patients is yet unknown. On these basis, we performed a phase I study to find the biologically "adequate" dose of magnesium valproate that would induce achieve histone acetylation and inhibition of HDAC in tumors of patients with cervical cancer.

## Results

### Study group

A total of 12 patients were studied. All of were chemotherapy- or radiation-naive and had a macroscopic tumor accessible for punch biopsy. Patient mean age was 63.3 years (44–72 years), while the majority of cases were squamous histology and were staged as FIGO stages IIB and IIIB. Status performance was 0–1 in most patients (Table 1).

**Table 1:** Clinical characteristics of patients

Number	12
Mean age (years)	63.3 (72–44)
Histology	
Squamous	8 (66%)
Adenocarcinoma	4 (33%)
FIGO stage*	
IIB	3 (25%)
IIIB	8 (66%)
IVB	1 (08%)
Performance status**	
0	1 (17%)
1	9 (75%)
2	2 (08%)

*FIGO = International Federation of Gynecology and Obstetrics; ** World Health Organization (WHO) criteria.

### Treatment compliance and side effects

All patients completed the study medication. Weight of patients varied from 45–82 kg and mean daily dose for all patients was 1,890 mg. According to dose level, mean daily dose for the 20-mg/kg dose level was 1245 mg (range, 1000–1400 mg), while it was 2000 mg (1800–2100) for the 30 mg/kg- and 2425 mg (1800–3300) for the 40-mg/kg dose level. Treatment in general was well-tolerated. Table 2 shows toxicity recorded according to the Common Toxicity Criteria (CTC). As expected, the most common side effect was depressed level of consciousness that in no case was grade 3 or 4. Thus, three of four patients at the 20-mg and 30-mg dose levels presented this event graded as 2, whereas one and three patients were graded 1 and 2, respectively, at the 40-mg dose; by definition, these side effects did not interfere with the daily living activities of patients. The next effect in frequency was fatigue; also grade 2 in three patients at the 20-mg dose level and in one patient at the 30-mg dose. Other side effects such as nausea, diarrhea, anorexia, and dizziness/lightheadedness were uncommon and mild. There were no changes in the values of non-hematological or hepatic parameters except by lymphopenia grade 1 in a patient receiving the lowest dose level. All toxicities disappeared within the ensuing week.

**Table 2:** Toxicity to valproate expressed by number of patients suffering the event

Toxicity	4 patients at each dose level (mg/kg)		
	20	30	40
Depressed level of consciousness*	3 (g2)	3(g2)	1(g1), 3(g2)
Nausea	-	1 (g1), 1(g2)	1 (g2)
Diarrhea	-	-	-
Fatigue	3 (g2)	1(g2)	-
Anorexia	-	1(g2)	-
Dizziness/lightheadedness	-	1(g2)	-

*Grade 2 toxicity. All others were grade 1.

Depressed level of consciousness (g1: somnolence or sedation not interfering with function; g2: somnolence or sedation interfering with function but not interfering with activities of daily living; g3: obtundation or stupor, difficulty to arouse, interfering with daily living; g4) coma.

### Histone acetylation of tumors

**Figure 1:**
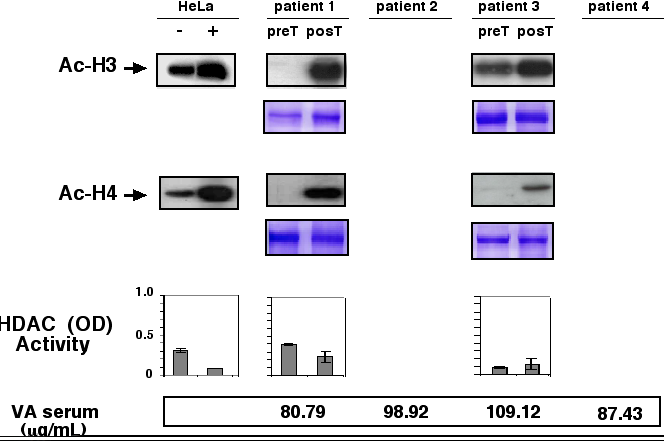
Western blots for anti-acetylated H3 and H4 histones pre- and post-treatment in patients receiving a dose of 20 mg/kg. Patients 2 and 4 are missed due to insufficient sample. Positive and negative controls are HeLa cells treated or not with trichostatinA. Loading control are gels stained with coomassie blue. Graphs in the inferior panel are HDAC activity expressed as optical densities (ODs), in the same scale as positive and negative controls that are also HeLa cells extracts with and without trichostatinA treatment. At the bottom are values of valproic acid in serum.

**Figure 2:**
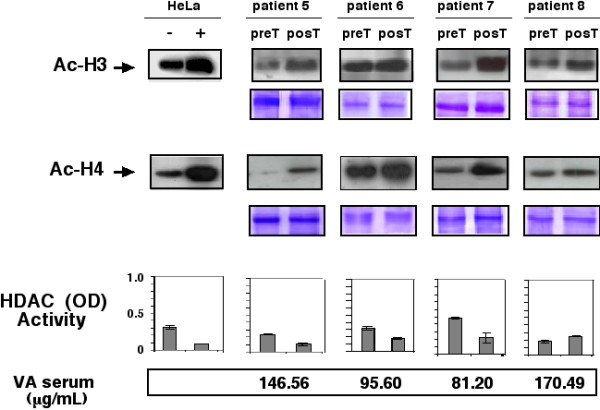
Western blots for anti-acetylated H3 and H4 histones pre- and post-treatment in patients receiving a dose of 30 mg/kg. Positive and negative controls are HeLa cells treated or not with trichostatin A. Loading control are gels stained with coomassie blue. Graphs in the inferior panel are HDAC activity expressed as ODs, in the same scale as positive and negative controls that are also HeLa cells extracts with and without trichostatinA treatment. At the bottom are values of valproic acid in serum.

**Figure 3:**
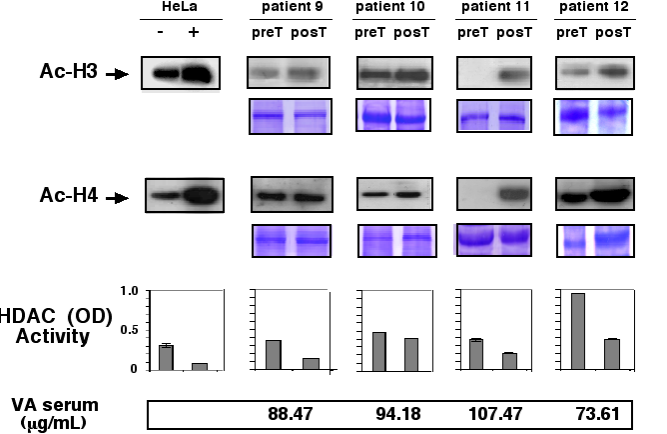
Western blots for anti-acetylated H3 and H4 histones pre- and post-treatment in patients receiving a dose of 40 mg/kg. Positive and negative controls are HeLa cells treated or not with trichostatin A. Loading control are gels stained with coomassie blue. Graphs in the inferior panel are HDAC activity expressed as ODs, in the same scale as the positive and negative controls that are also HeLa cells extracts with and without trichostatin A treatment. At the bottom are values of valproic acid in serum.

Pre- and post-treatment tumor samples of all 12 patients were collected; however, the effect of valproate treatment on histone acetylation by Western blot of H3 and H4 could not be assessed in two patients because amount and quality of tumor samples of either pre- or post-treatment biopsies were not adequate. These two patients (numbers 2 and 4) belonged to the 20-mg/kg dose level. Figures 1, 2, and 3 are Western blots of the patients analyzed. The first observation is the ample heterogeneity in degree of baseline acetylation in both H3 and H4 histones. Pre-treatment band was hardly seen for acetylated H3 in patients 1 and 11, whereas it was very strong in patient 6; likewise, acetylated H4 was very weak in patients 3, 5, and 11. In assessment of valproate treatment effect, there was variable increase in band intensity, indicative of hyperacetylation of H3 in all patients except in patient 6. With regard to acetylation of H4, seven patients (1, 3, 5, 6, 7, 11, and 12) had clear hyperacetylation, whereas in the remaining individuals (patients 8, 9, and 10) the effect was minor or non-existent. As can be seen, both H3 and H4 hyperacetylation was observed in patients 1, 3, 5, 7, 11 and 12.

### Serum Levels of valproic acid

Blood serum levels of valproic acid after the 5-day treatment period are shown in Figures 1, 2, and 3, respectively. Samples were taken and analyzed in all cases. Levels ranged from 73.6–170.49 μg/mL. There was lack of correlation between serum levels with dose level. Thus, values for patients were as follows: at the 20-mg/kg dose level 80.79, 98.92, 109.12, and 87.43, for a mean of 94.06 μg/mL; for the 30-mg/kg level 146.56, 81.20, 170.49, and 95.60, for a mean of 123.46 μg/mL, and finally for the highest dose level of 40 mg/kg, corresponding values were 88.47, 94.18, 107.47, and 73.61, with a mean of 90.93 μg/mL.

### Histone deacetylase assay in tumors

To investigate whether a decrease in histone acetylase activity could be achieved by valproate treatment in the tumors, enzyme activity was evaluated in tumor biopsies extracts by colorimetric commercial HDAC activity assay in 10 patients; Results are also shown in Figures 1, 2, and 3. Using the same scale for positive and negative control, we found that 8 of 12 (75%) patients had a decrease in deacetylase activity equal to or greater that observed in HeLa cell extracts treated or not with trichostatinA, whereas patients 3 and 8 (25%) had either no change or a mild increase in deacetylase activity. Pooling the absolute values of optical densities (ODs) for pre- and post-treatment samples, there was a statistically significant difference between values (mean 0.36 vs. 0.21, respectively, for a 0.142 mean difference 95% confidence interval (CI 95%) 0.020–0.264), two-tailed *t *test (*p *< 0.0264). Our small sample size did not allow for establishing a correlation with H3 and H4 histone acetylation. Remarkably, cases 3 and 8-who showed no HDAC inhibition had tumor hyperacetylation. The patient 3 in both histones whereas the patient 8 only in H3.

### H3 and H4 Acetylation in PBMN cells

**Figure 4:**
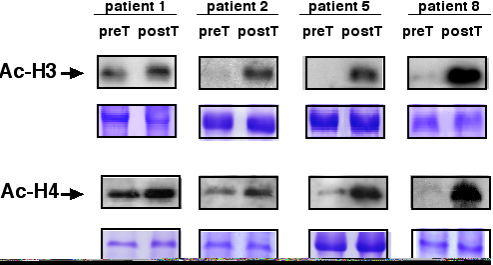
Western blots for anti-acetylated H3 and H4 histones pre- and post-treatment in PBMN cells of patients 1, 2, 5, and 8. Patient 1 and 2 received a dose of 20 mg/kg, whereas patients 5 and 8 each received a dose of 30 mg/kg. Positive and negative controls are HeLa cells treated or not with thrichostatin A. Loading control are gels stained with Coomassie blue.

Due to limitations in amount of blood samples, histone acetylation by Western blot could be assayed only in four patients (1, 2, 5, and 8). Figure 4 shows that all four showed hyperacetylation after treatment in comparison with pre-treatment samples for both H3 and H4 histones.

## Discussion

The balance between histone deacetylases and histone acetyl transferase activities is a major player in regulation of gene transcription [19]. HDAC inhibition induces accumulation of hyperacetylated nucleosome core histones in the majority of chromatin regions but affects expression of only a small subset of genes, leading to transcriptional activation of some but repression of an equal or larger number of other genes [20,21]. This led to testing HDAC inhibitors as anticancer agents in a variety of tumor models and clinical studies. To date, a number of structurally distinct classes of compounds have been identified as HDAC inhibitors including hydroxamates, cyclic tetrapeptides, benzamides, and short-chain fatty acids [22].

The discovery that valproic acid-which belongs to the short-chain fatty acids category and resulted to be an effective HDAC inhibitor [17,18]-encouraged investigation of this agent as a potential cancer therapy agent. Valproic acid can be administered as such or as sodium or magnesium salts, but all three forms have the same pharmacokinetic behaviour and anticonvulsant effect [23]. Here we demonstrate that magnesium valproate, when used at only slightly higher doses than those used as anticonvulsant, not only produces H3 and H4 histone hyperacetylation in PBMN cells but also leads to hyperacetylation of H3 and H4 and inhibition of HDAC activity in tumors.

Preclinical data has shown that valproic acid inhibits the majority of class I and II HDAC at CI50, varying from 0.7–1.3 mM [24]; however, the concentration needed *in vitro *to achieve its biological effects varies from system to system, but in some cases the concentration required is lower than 0.5 mM in tumors such as glioma, hematopoietic, or breast cancer cell lines [24-26]. On the other hand, it is also known that the inhibitory effect of valproic acid on HDAC begins and disappears within hours after exposure, with a rapid return of baseline acetylation on histones [24]. This knowledge suggests that valproic acid, for effective use in the clinical setting, should be administered continuously and perhaps for long periods. Nonetheless, despite the fact that the therapeutic window of valproic acid is very wide (50–100 μg/mL), there is a clear dose-effect relationship with depressed level of consciousness [27] therefore we felt important to investigate if doses below 60 mg/Kg which produce grade III toxicity lead to histone hyperacetylation in tumors.

These facts led to us to perform this phase I study to find the "adequate" biological dose of magnesium valproate. The advent of the so called "targeted therapies" has led to reconsider the appropriateness of the traditional or classical designs for phase I trials which takes into account toxicity as the most important parameter for dose escalation. Instead some authors have proposed that the endpoint in phase I studies of targeted therapies should be a change in the level or activity of the target or enzyme or other surrogate marker [28]. Because it is clear that the antiproliferative, differentiating and/or pro-apoptotic effects of HDAC inhibitors result from the histone hyperacetylation it seems logical to evaluate the acetylation status of H3 and H4 histones as the endpoint of the trial. On the other hand, not necessarily, the highest hyperacetylation should occur at the higher dose of the tested agent, being possible that a plateau in the effect can be seen in a range of doses that do not produce limiting toxicity. In this sense, the concept of "adequate" instead of the "optimal" biological dose emerges because, by one hand, the number of patients required for finding the optimal dose may be to high for an agent that by itself (or as a single agent) is expected to produce a negligible clinical response rate or tumor shrinkage which equate to expose a larger number of patients to a potentially ineffective agent by its own [29,30]. Another issue for selecting adequate instead of optimal dose design is the fact that it allows investigators to quickly proceed to phase II trials (usually along with chemotherapy or radiation). Perhaps the only situation where an optimal dose design is preferred accounts when the study drug is planned to proceed to larger phase III trials [31].

On this basis, we opted to perform an "adequate" dose phase I. To our surprise, using doses as low as 20–40 mg/kg, the biological effect was observed, being remarkable that no patient presented any grade 3 event. In nine patients, depressed level of consciousness was grade 2. Molecular efficacy in terms of H3 and H4 hyperacetylation in tumors was observed in most patients; in fact if we take into account the effects on H3, at 20 mg/Kg 2 out of 2 evaluated (100%) had hyperacetylation, 3 out of 4 at 30 mg/Kg (75%), and 4 out of 4 (100%) patients at 40 mg/Kg. For H4 a very similar pattern emerged 2/2 (100%), 3/4 (75%) and 2/4 (50%) had hyperacetylation at 20 mg, 30 mg and 40 mg/Kg. When evaluating for both H3 and H4 acetylation the numbers are 2/2 (100%), 2/4 (50%), and 2/4 (50%). Overall, despite the limited number of patients, inherent to the study design it is clear that at the range of doses tested there is a plateau in the molecular response (100%, 50% and 50%) for the three tested doses. In cases like this when there is a tie in efficacy, the highest dose should be selected, and hence we have selected the dose of 40 mg/Kg for our phase II studies [29].

Currently, there is no information concerning the effect of HDAC inhibitors upon acetylation of H3 and H4 in solid tumors of patients. Two reports on depsipeptide have demonstrated hyperacetylation of PBMN cells in two patients with cutaneous T-cell lymphoma and two patients with refractory neoplasms as evaluated by immunoflouorescent labelling [32,33]; additionally, in a clinical study of SAHA for lymphoma and solid tumors it was reported that three of five patients showed increased accumulation of acetylated H3 in the post-treatment biopsy sample [34]. These data are in agreement with our findings indicating that the hyperacetylating effect is clinically achievable with these HDAC inhibitors.

The inhibitory activity of HDAC in cells shown by this class of compounds always parallels changes in H3 and H4 acetylation [24]. We demonstrate herein that valproic acid produces a decrease in HDAC activity in tumors which is accompanied by histone hyperacetylation suggesting that the H3 and H4 hyperacetylation occurring in tumors is a consequence of HDAC inhibition. On the other hand, it is remarkable that three out of the four cases assayed by H3 and H4 acetylation by western blot in PBMN cells (patients 1, 5 and 8) their corresponding tumors had hyperacetylation showing the concordance between the findings in tumors and PBMN cells. These data suggest that HDAC activity in the tumor or H3 and H4 acetylation status in the PBMN cells can be good surrogate markers for monitoring the effect of HDAC inhibitors in clinical trials.

The pharmacokinetics of valproic acid has extensively been studied. Because its half-life is between 8–16 hours, the steady-state concentrations can be determined as soon as at day 4 of treatment [35,36]. Under this rational we determined the levels of valproic acid at day six, within 8–10 hours of the last dose taken, hence the sera values obtained are representative of the levels at the steady-state. High interindividual variation in the pharmacokinetics of valproic acid in epileptic patients is well-known [37]. This phenomenon was observed in our study with serum concentrations ranging from 73.61 to 170.49 μg/mL, and in addition there was no correlation with dose level and mean concentration achieved; thus, for doses of 20, 30, and 40 mg/kg, mean concentrations were 94.06, 123.46, and 90.93 μg/mL, respectively. This finding, however, could be derived from the small number of patients we analyzed, because Tisdale et al. demonstrated significant linear correlation between VPA dose and serum concentration in a sample of 60 epileptic patients [37]. Alternatively, it can be suggested that like in cell lines, which show high variability in response to HDAC inhibitors [24-26], interpatient variability in tumors may exist.

There is one report in abstract form proceeding from a clinical study with valproic acid. Atmaca et al., performed a "traditional" dose escalating phase I study using valproic acid by intravenous infusion in patients with advanced cancer. In the study, 26 patients pre-treated and with progressive malignant disease received the drug by 1-h infusion split twice a day for 5 consecutive days, repeating the treatment at intervals of 2 weeks in cohorts of 60, 75, 90, and 120 mg/kg dosages. These investigators found maximum tolerated dose of 60 mg/kg because 9 of 26 patients presented grade 3/4 neurological toxicity. In addition, these authors reported hyperacetylation of PBMN cells in the majority of patients; nevertheless, they provided no information on serum levels achieved or the molecular response according to the dose level [38].

In a phase I-II study of the combination of decitabine and valproic acid for myeloblastic acute leukemia and myelodysplastic syndrome, a fixed dose of 15 mg/m2 of decitabine and valproic acid at 20, 35 and 50 mg/Kg were administered every 10 days. The toxicity observed was grade III neurotoxicity at 50 mg/Kg in 1 of 10 and grade II in 5 out 10 patients. Subsequently, VPA 50 mg/kg was chosen for the phase II portion of the study. Of 40 evaluable for response, an overall response rate of 22% (8 PR and 1 CR) was observed. Interestingly, the response rates according to the valproic acid dose were 33% for 20 mg, 11% for the 35 mg and 25% for the 50 mg/Kg dose level [39]. Finally, in a third phase I study for metastatic solid tumors valproic acid was administered as an IV loading dose followed by 5 doses of oral doses given every 12 hours followed by a dose of epirubicin at day 3. At the time of reporting, 16 patients have been treated at 4 dose levels: VPA 15, 30, 45, and 60 mg/kg; with epirubicin at 75 mg/m^2^. The maximum tolerated dose has not been reached and dose escalation is continuing. Major responses were observed in all tumor types including in anthracycline failures and in anthracycline-resistant cancers such as melanoma and cervical carcinoma. Plasma levels were not reported, however, there were above the concentrations needed for in-vitro synergy [40].

The extensive use of valproate as anticonvulsant has shown excellent tolerability in the therapeutic range between 50 and 100 μg/mL [15,16]. Despite the fact that levels >100 μg/mL are considered supra-therapeutic [41], it must be stressed that in the our present study four patients presented concentrations higher than that level (109.12, 146.56, 170.49, and 107.43 μg/mL), and that none of these patients presented any grade 3 toxicity, which indicates the feasibility of achieving these levels during treatment to maximize the chances of producing tumor hyperacetylation. In fact, significant clinical adverse effects of valproic acid ingestion, such as lethargy, coma, tachycardia, metabolic acidosis, and hypotension, are more likely to occur with concentrations >450 μg/mL [41].

All together, existing data suggest that molecular and clinical responses occur at valproic acid doses between 20 and 60 mg/Kg. The optimal biological dose of valproic acid remains to be determined, however, our results suggest the existence of a plateau in the histone acetylation at doses between 20 and 40 mg/Kg. Based on that we have chosen 40 mg/Kg as the dose to test in phase II trials.

Synergy of HDAC inhibitors with demethylating agents for reactivating expression of tumor suppressor genes as well as to induce antitumor effects is well-known [42-44]. In this regard, our group showed that hydralazine is an effective demethylating agent that reactivates the tumor suppressor genes expression silenced by methylation [45,46] and reported the results of a phase I study demonstrating the gene demethylating and reactivating activity of the drug at doses between 75 and 150 mg/day [47]. On these bases, we at present are testing these two drugs-demethylating hydralazine and the HDAC inhibitor valproic acid-plus chemotherapy or plus chemoradiation in phase II studies for solid tumors.

## Conclusion

Our results provide evidence that magnesium valproate at doses between 20 mg and 40 mg/Kg, inhibits deacetylase activity and hyperacetylates histones in tumor tissues. Its clinical efficacy along with a demethylating agent plus chemotherapy or radiation is currently being tested in phase II studies.

## Methods

### Patient selection

Previously untreated patients with histological diagnosis of carcinoma of the cervix uteri entered into this phase I study. Patients were invited to participate in the study in the waiting time from diagnostic evaluation to commencing chemoradiation. The whole study period lasted only 6 days, hence valproic acid treatment was not repeated.

The following inclusion criteria were applied: 1) age between 18 and 75 years; 2) World Health Organization Performance Status 0–2; 3) hematological, renal, and hepatic functions as follows: hematological: hemoglobin ≥10 g/L; leukocytes >4,000/mm^3^, platelets >100,000/mm^3^, total bilirubin and transaminasas <1.5× the normal upper limit, and normal serum creatinine; 4) normal chest x-ray, and 5) signed informed consent for study medication and biopsies pre and post-treatment. Exclusion criteria included the following: 1) history of allergy to valproic acid; 2) any past or current central nervous system pathologic condition (epilepsy, etc.) that required pharmacologic treatment; 3) any current hepatic disease; 4) uncontrolled infection or other systemic diseases; 5) concomitant treatment with any experimental drug; 6) pregnant or nursing women; 7) mental illness, and 8) previous or concomitant malignant diseases other than non-melanoma skin cancer. The Institutional Regulatory Board approved the study protocol.

### Clinical samples

Biopsies were taken from areas with visible macroscopic cervical tumor using a sterile biopsy punch the day before and the day six after the five days of magnesium valproate treatment. (The post-treatment biopsy and peripheral blood sampling were taken in the early morning, between 8 and 10 hours after the last dose of magnesium valproate). Part of the biopsy was sent to the Institution's Pathology Department for routine Hematoxilin & eosin diagnosis. The remaining biopsy specimen was immediately frozen at -20°C for biological analyses. In addition, a blood sample of 10 mL was drawn from the arm by venipuncture to obtain serum and the mononuclear cell fraction for protein extraction.

### Magnesium valproate treatment

After tumor and blood sampling, patients were divided into the following groups and started oral magnesium valproate for a five-day period: I) 20 mg/kg, II) 30 mg/kg; III) 40 mg/Kg. Total dose was divided in three administrations every 8 h (8 AM, 4 PM and 12 PM) per oral route in enteric-coated tablets of 200 and/or 400 mg. Biological effects of treatment on tumor and peripheral blood samples as well as clinical and laboratory toxicity were assessed at the end of the study period (6 days). Patients then went to receive the definitive chemoradiation treatment as planned.

Toxicity was registered according to the Common Toxicity Criteria of the National Cancer Institute (CTC NCI) with special emphasis on the presence, throughout the treatment days, of known signs and symptoms associated with magnesium valproate treatment such as somnolence/sedation and fatigue. Therapeutic levels of valproic acid were also evaluated in blood serum at day 6, between 8 and 10 hours after the last dose of magnesium valproate.

### Acid extraction of proteins

Acid extraction of histones was performed as described previously [48] with modifications. Briefly, tumor samples and mononuclear cell pellets were suspended in 10 volumes of PBS and centrifuged at 200 × *g *for 10 min. Cells were then suspended with five volumes of lysis buffer [10 mM HEPES (pH 7.9), 1.5 mM MgCl_2_, 10 mM KCl, 0.5 mM DTT, and 1.5 mM phenylmethylsulfonyl fluoride] and hydrochloride acid at a final concentration of 0.2 M and subsequently lysed on ice for 30 min. After centrifugation at 11,000 × *g *for 10 min at 4°C, the cell supernatant fraction that contained acid-soluble proteins was retained. Supernatant was dialyzed against 200 mL of 0.1 M acetic acid twice for 1–2 h each and then dialyzed against 200 mL of H_2_O for 1 h, 3 h, and overnight. Dialysis was performed using a Spectra/Pore 3 Dialysis Membranes 3,500 MWCO (Spectrum Laboratories, Inc., Rancho Dominguez, CA, USA).

### Western blot analysis

Acid extracted proteins were analyzed by sodium duodecyl sulfate-polyacrylamide gel electrophoresis (SDS-PAGE)/immunoblotting with antibodies recognizing acetylated histones (rabbit polyclonal IgG, anti-acetyl-histone H4, and rabbit polyclonal-anti-acetyl-histone H3; Upstate Biotechnology, Lake Placid, NY, USA). Protein samples were separated along with molecular weight markers (Bio-Rad, Hercules, CA, USA) in 15% polyacrylamide gels. Gels were transferred onto 0.45-μm nitrocellulose membranes (Schleicher and Schuell, Keene, NH, USA). Gel loading equivalence was confirmed by Coomassie blue stain (Sigma, St Louis, MO, USA). Species-specific immunoglobulin G-horseradish peroxidase (IgG-HRP) secondary antibodies were purchased from Bio-Rad. Blots were developed with chemiluminescent substrate (Amersham International, Buckinghamshire England), and autoradiography was performed utilizing X-OMAT film (Kodak, Rochester, NY, USA).

### Histone deacetylase assay

Assays were performed using the colorimetric HDAC activity assay from BioVision (BioVision Research Products, Mountain View, CA, USA) according to manufacturer instructions. Briefly, 50 μg of nuclear extracts from tumors were diluted in 85 μL of ddH_2_O; then, 10 μL of 10× HDAC assay buffer were added followed by addition of 5 μL of the colorimetric substrate; samples were incubated at 37° for 1 h. Subsequently, the reaction was stopped by adding 10 μL of lysine developer and left for additional 30 min at 37°C. Samples were then read in an ELISA plate reader at 400 nm. HDAC activity was expressed as relative OD values per μg of protein sample. The kit contains negative and positive controls that consist of nuclear extract of HeLa treated or not with trichostatinA, respectively.

Blood levels of valproic acid. Valproic acid was measured in serum using a fluorescence polarization immunoassay (FPIA) technology. This is a competitive binding immunoassay technology in which fluorescein-labeled and patient antigens compete for binding sites on antibody molecules [49]. Polarization of the fluorescent light emitted from labeled drug-antibody complex is inversely related with amount of drug in the sample. TDx/TDxFLx analyzer and the valproic acid kit REF9514 were obtained from Abbott Laboratories (Abbott Laboratories, Abbott Park, IL, USA). Blood samples were obtained in an sst-gel-clot activator tube 8 h after the last dose of magnesium valproate and assays were conducted following manufacturer instructions; 100-μL samples of serum were run by duplicate and values expressed as μg/mL.

### Statistical analysis

All numerical data were expressed as average of values obtained ± standard deviation (SD). Statistical significance was determined by conducting a paired Student *t *test.

## Competing interests

The author(s) declare that they have no competing interests.

## Authors' contributions

PZ participated the collection and storing of clinical samples; A C-B and B S-P, performed the Western blots and histone deacetylase assays, A G-F and P G-L participated in the determination of serum valproic acid levels; E P-C and L T-C participated in analysis of results, LC, MC and DC cared for patients, EA, GC, CP-P and CT-B participated in discussion of results, A D-G conceived of the study and wrote the manuscript.
